# Supplementary figures and images for: Increased tooth brushing frequency is associated with reduced gingival pocket bacterial diversity in patients with intracranial aneurysms
Source: PeerJ. 2019 Jan 25;7:e6316. doi: 10.7717/peerj.6316 (PMC6348950; doi:10.7717/peerj.6316)

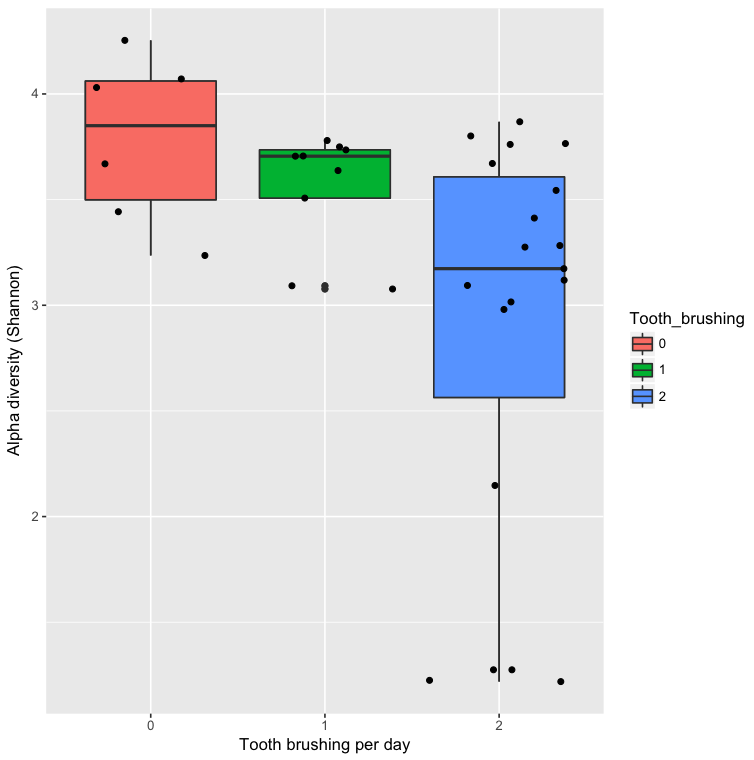

Supplement: Supplemental Information 2 [file peerj-07-6316-s002.png]

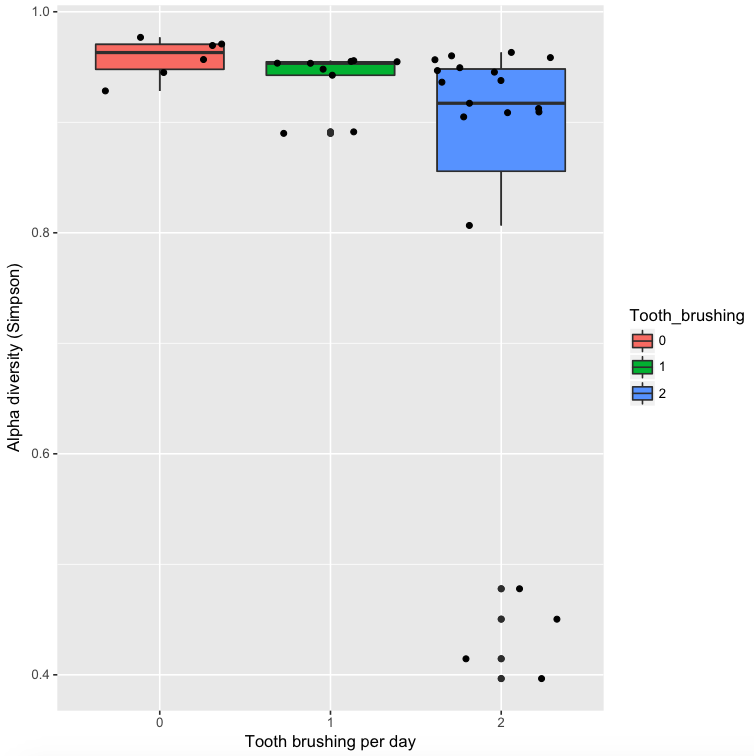

Supplement: Supplemental Information 3 [file peerj-07-6316-s003.png]
