# Supplementary material for: Increased tooth brushing frequency is associated with reduced gingival pocket bacterial diversity in patients with intracranial aneurysms
Source: PeerJ. 2019 Jan 25;7:e6316. doi: 10.7717/peerj.6316 (PMC6348950; doi:10.7717/peerj.6316)
Supplement: Supplemental Information 4 — Coding done for the analyses. [file peerj-07-6316-s004.pdf]

/wrk/mishra/sav

```
gunzip sav_data/*.gz
```

Check quality of all the files

```
chmod 0777 ./FastQC/fastqc (if permission denied)
./FastQC/fastqc sav_data/*.fastq
```

Remove primers

forward

```
for i in $(ls /wrk/mishra/sav/sav_data/*.fastq)

do

python cutadapt -b CCTACGGGAGGCAGCAG -o ${i%}.noprimer ${i%}

done
```

```
# Remove the old files

rm sav_data/*.fastq

# remove “noprimer” suffix from file names

for file in sav_data/*.noprimer; do
   mv -- "$file" "${file%%.noprimer}"
done
```

reverse

```
for i in $(ls /wrk/mishra/sav/sav_data/*.fastq)

do

python cutadapt -b GGACTACCAGGGTATCTAAT -o ${i%}.noprimer ${i%}

done
```

Remove the old files

```
rm sav_data/*.fastq

for file in sav_data/*.noprimer; do
   mv -- "$file" "${file%%.noprimer}"
done
```

Trim bad quality sequence.

```
for f1 in *_R1_001.fastq
do
    f2="${f1/R1/R2}"
    trimmomatic PE -threads 8 -phred33 $f1 $f2 ${f1}
_paired.fastq ${f1}_unpaired.fastq ${f2}_paired.fastq ${f2}
_unpaired.fastq ILLUMINACLIP:/appl/bio/trimmomatic/adapters/
TruSeq3-PE.fa:2:30:10 LEADING:3 TRAILING:3 SLIDINGWINDOW:4:15
MINLEN:200
done


# remove unpaired ones
rm -rf *_unpaired.fastq

# move trimmed data to another folder
mkdir trimmed_data
mv *_paired.fastq trimmed_data/
```

Quality check of trimmed samples

```
./FastQC/fastqc sav_data/trimmed_data/*.fastq
```

Remove the '_paired.fastq' suffix from the file names

```
for file in *_paired.fastq; do
    mv -- "$file" "${file%%_paired.fastq}"
done
```

Merge paired ends in

```
for i in $(ls *.fastq | rev | cut -c 13- | rev | uniq)
do
join_paired_ends.py -f ${i}R1_001.fastq -r ${i}R2_001.fastq -o
merged${i}
done


# remove unmerged files (separate file haru)
rm -rf *.fastq
```

Rename the funny merged file names by qiime

```
for d in */ ; do
    x=$(echo $d | grep -o -P '(?<=merged).*(?=L001)')
    z=$(echo "${x%?}")
    mv ${d%}/fastqjoin.join* ${z%}.fastq
done
```

convert all the files into fasta from fastq

```
for i in $(ls *fastq I rev I cut -c 7- I rev I uniq)
do
paste - - - - < ${i}.fastq I sed 's/^@/>/g'I cut -f1-2 I tr '\t' '\n' > ${i}.fasta
done
```

number of seqs in files (useful for normalization)

```
count_seqs.py -i "*.fasta"
```

Until now 11 samples excluded due to low coverage and 2 were excluded because of bad quality.

Contamination filtration based on duplicates here.

```
assign_taxonomy.py -i mydata/18a-1910_S28.fasta -r
HOMD_16S_rRNA_RefSeq_V14.51.aligned.fasta -t
HOMD_16S_rRNA_RefSeq_V14.5.qiime.taxonomy  -o taxonomy_18a
assign_taxonomy.py -i mydata/18b-1910_S29.fasta -r
HOMD_16S_rRNA_RefSeq_V14.51.aligned.fasta -t
HOMD_16S_rRNA_RefSeq_V14.5.qiime.taxonomy  -o taxonomy_18b

assign_taxonomy.py -i mydata/19a-1910_S30.fasta -r
HOMD_16S_rRNA_RefSeq_V14.51.aligned.fasta -t
HOMD_16S_rRNA_RefSeq_V14.5.qiime.taxonomy  -o taxonomy_19a
assign_taxonomy.py -i mydata/19b-1910_S31.fasta -r
HOMD_16S_rRNA_RefSeq_V14.51.aligned.fasta -t
HOMD_16S_rRNA_RefSeq_V14.5.qiime.taxonomy  -o taxonomy_19b


assign_taxonomy.py -i mydata/20a-1910_S32.fasta -r
HOMD_16S_rRNA_RefSeq_V14.51.aligned.fasta -t
HOMD_16S_rRNA_RefSeq_V14.5.qiime.taxonomy  -o taxonomy_20a
assign_taxonomy.py -i mydata/20b-1910_S33.fasta -r
HOMD_16S_rRNA_RefSeq_V14.51.aligned.fasta -t
HOMD_16S_rRNA_RefSeq_V14.5.qiime.taxonomy  -o taxonomy_20b


assign_taxonomy.py -i mydata/K3a-1910_S34.fasta -r
HOMD_16S_rRNA_RefSeq_V14.51.aligned.fasta -t
HOMD_16S_rRNA_RefSeq_V14.5.qiime.taxonomy  -o taxonomy_K3a
assign_taxonomy.py -i mydata/K3b-0211_S51.fasta -r
HOMD_16S_rRNA_RefSeq_V14.51.aligned.fasta -t
HOMD_16S_rRNA_RefSeq_V14.5.qiime.taxonomy  -o taxonomy_K3b
```

```
# 18
taxa_18a <- unique(as.character(read.table("18a-1910_S28_tax_assignments.txt")[,
2]))
taxa_18b <- unique(as.character(read.table("18b-1910_S29_tax_assignments.txt")[,
2]))
intersect_18 <- intersect(taxa_18a,taxa_18b)
unq_18a <- taxa_18a[!taxa_18a %in% intersect_18] # taxa unique to samples
unq_18b <- taxa_18b[!taxa_18b %in% intersect_18]
ind_18a <-
match(unq_18a,as.character(read.table("18a-1910_S28_tax_assignments.txt")[,2]))
ind_18b <-
```

```
match(unq_18b,as.character(read.table("18b-1910_S29_tax_assignments.txt")[,2]))
id_18a <- as.character(read.table("18a-1910_S28_tax_assignments.txt")[ind_18a,
1]) # ids of taxa to be removed
id_18b <- as.character(read.table("18b-1910_S29_tax_assignments.txt")[ind_18b,
1])
write.table(id_18a,file="id_18a.txt",quote=FALSE,row.names=FALSE)
write.table(id_18b,file="id_18b.txt",quote=FALSE,row.names=FALSE)

Module load biokit
./extractById.py /wrk/mishra/sav/mydata/18a-1910_S28.fasta id_18a.txt
18a_bad_seqs.fasta # extract sequences to be removed
./extractById.py /wrk/mishra/sav/mydata/18b-1910_S29.fasta id_18b.txt
18b_bad_seqs.fasta
usearch -usearch_global  /wrk/mishra/sav/mydata/18a-1910_S28.fasta -db
18a_bad_seqs.fasta -id 0.99 -strand plus --notmatched filtered_18a.fasta -threads 2
usearch -usearch_global  /wrk/mishra/sav/mydata/18b-1910_S29.fasta -db
18b_bad_seqs.fasta -id 0.99 -strand plus --notmatched filtered_18b.fasta -threads 2
```

# 19

```
taxa_19a <- unique(as.character(read.table("19a-1910_S30_tax_assignments.txt")[,
2]))
taxa_19b <- unique(as.character(read.table("19b-1910_S31_tax_assignments.txt")[,
2]))
intersect_19 <- intersect(taxa_19a,taxa_19b)
unq_19a <- taxa_19a[!taxa_19a %in% intersect_19] # taxa unique to samples
unq_19b <- taxa_19b[!taxa_19b %in% intersect_19]
ind_19a <-
match(unq_19a,as.character(read.table("19a-1910_S30_tax_assignments.txt")[,2]))
ind_19b <-
match(unq_19b,as.character(read.table("19b-1910_S31_tax_assignments.txt")[,2]))
id_19a <- as.character(read.table("19a-1910_S30_tax_assignments.txt")[ind_19a,
1]) # ids of taxa to be removed
id_19b <- as.character(read.table("19b-1910_S31_tax_assignments.txt")[ind_19b,
1])
write.table(id_19a,file="id_19a.txt",quote=FALSE,row.names=FALSE)
write.table(id_19b,file="id_19b.txt",quote=FALSE,row.names=FALSE)

module load biokit
./extractById.py /wrk/mishra/sav/mydata/19a-1910_S30.fasta id_19a.txt
19a_bad_seqs.fasta # extract sequences to be removed
./extractById.py /wrk/mishra/sav/mydata/19b-1910_S31.fasta id_19b.txt
19b_bad_seqs.fasta
usearch -usearch_global  /wrk/mishra/sav/mydata/19a-1910_S30.fasta -db
19a_bad_seqs.fasta -id 0.99 -strand plus --notmatched filtered_19a.fasta -threads 2
usearch -usearch_global  /wrk/mishra/sav/mydata/19b-1910_S31.fasta -db
19b_bad_seqs.fasta -id 0.99 -strand plus --notmatched filtered_19b.fasta -threads 2
```

# 20

taxa_20a <- unique(as.character(read.table("20a-1910_S32_tax_assignments.txt")[,
2]))
taxa_20b <- unique(as.character(read.table("20b-1910_S33_tax_assignments.txt")[,
2]))
intersect_20 <- intersect(taxa_20a,taxa_20b)
unq_20a <- taxa_20a[!taxa_20a %in% intersect_20] # taxa unique to samples
unq_20b <- taxa_20b[!taxa_20b %in% intersect_20]
ind_20a <-
match(unq_20a,as.character(read.table("20a-1910_S32_tax_assignments.txt")[,2]))
ind_20b <-
match(unq_20b,as.character(read.table("20b-1910_S33_tax_assignments.txt")[,2]))
id_20a <- as.character(read.table("20a-1910_S32_tax_assignments.txt")[ind_20a,
1]) # ids of taxa to be removed
id_20b <- as.character(read.table("20b-1910_S33_tax_assignments.txt")[ind_20b,
1])
write.table(id_20a,file="id_20a.txt",quote=FALSE,row.names=FALSE)
write.table(id_20b,file="id_20b.txt",quote=FALSE,row.names=FALSE)

module load biokit
./extractById.py /wrk/mishra/sav/mydata/20a-1910_S32.fasta id_20a.txt
20a_bad_seqs.fasta # extract sequences to be removed
./extractById.py /wrk/mishra/sav/mydata/20b-1910_S33.fasta id_20b.txt
20b_bad_seqs.fasta
usearch -usearch_global /wrk/mishra/sav/mydata/20a-1910_S32.fasta -db
20a_bad_seqs.fasta -id 0.99 -strand plus --notmatched filtered_20a.fasta -threads 2
usearch -usearch_global /wrk/mishra/sav/mydata/20b-1910_S33.fasta -db
20b_bad_seqs.fasta -id 0.99 -strand plus --notmatched filtered_20b.fasta -threads 2

# K3

taxa_K3a <- unique(as.character(read.table("K3a-1910_S34_tax_assignments.txt")[,
2]))
taxa_K3b <- unique(as.character(read.table("K3b-0211_S51_tax_assignments.txt")[,
2]))
intersect_K3 <- intersect(taxa_K3a,taxa_K3b)
unq_K3a <- taxa_K3a[!taxa_K3a %in% intersect_K3] # taxa unique to samples
unq_K3b <- taxa_K3b[!taxa_K3b %in% intersect_K3]
ind_K3a <-
match(unq_K3a,as.character(read.table("K3a-1910_S34_tax_assignments.txt")[,2]))
ind_K3b <-
match(unq_K3b,as.character(read.table("K3b-0211_S51_tax_assignments.txt")[,2]))
id_K3a <- as.character(read.table("K3a-1910_S34_tax_assignments.txt")[ind_K3a,

1]) # ids of taxa to be removed
id_K3b <- as.character(read.table("K3b-0211_S51_tax_assignments.txt")[ind_K3b, 1])
write.table(id_K3a,file="id_K3a.txt",quote=FALSE,row.names=FALSE)
write.table(id_K3b,file="id_K3b.txt",quote=FALSE,row.names=FALSE)

module load biokit
./extractById.py /wrk/mishra/sav/mydata/K3a-1910_S34.fasta id_K3a.txt K3a_bad_seqs.fasta # extract sequences to be removed
./extractById.py /wrk/mishra/sav/mydata/K3b-0211_S51.fasta id_K3b.txt K3b_bad_seqs.fasta
usearch -usearch_global /wrk/mishra/sav/mydata/K3a-1910_S34.fasta -db K3a_bad_seqs.fasta -id 0.99 -strand plus --notmatched filtered_K3a.fasta -threads 2
usearch -usearch_global /wrk/mishra/sav/mydata/K3b-0211_S51.fasta -db K3b_bad_seqs.fasta -id 0.99 -strand plus --notmatched filtered_K3b.fasta -threads 2

Move the filtered files to mydata/

# Now merge all samples.

add_qiime_labels.py -i mydata/ -m map_updated.txt -c InputFileName -n 1 -o combined_fasta

# side by side lets do analysis by removing bad quality samples (duplicates that are far)
add_qiime_labels.py -i mydata/ -m map_updated1.txt -c InputFileName -n 1 -o combined_fasta1

12. Chimera detection (usearch61)

#!/bin/bash -l
#SBATCH -J chimera
#SBATCH -o chimera.stdout
#SBATCH -e chimera.stderr
#SBATCH -n 1
#SBATCH -t 12:00:00
#SBATCH --nodes=1
#SBATCH --mem-per-cpu=23000

identify_chimeric_seqs.py -i combined_fasta/combined_seqs.fna -m usearch61 -o chimeric_seqs_blast --suppress_usearch61_ref

identify_chimeric_seqs.py -i combined_fasta1/combined_seqs.fna -m usearch61 -o

chimeric_seqs_blast1 --suppress_usearch61_ref

----

remove chimeric sequences

filter_fasta.py -f combined_fasta/combined_seqs.fna -o seqs_chimeras_filtered.fna -s chimeric_seqs_blast/chimeras.txt -n
filter_fasta.py -f combined_fasta1/combined_seqs.fna -o seqs_chimeras_filtered1.fna -s chimeric_seqs_blast1/chimeras.txt -n

Use mothur to remove mitochondrial, chloroplast, archaea and eukaryotes sequences.
The latest reference and taxonomy (11.01.2017) were downloaded from - https://www.mothur.org/wiki/RDP_reference_files. Check PDS vs RDP in the same link.
http://bioinformatics-ca.github.io/analysis_of_metagenomic_data_mod2_lab_2015/

a. Classify the sequences based on training dataset downloaded from mothur references. Here we use trainset9 instead of homd datasets because not sure what new datasets contain.

```
mothur > classify.seqs(fasta=seqs_chimeras_filtered.fna,
reference=trainset14_032015.pds.fasta,
taxonomy=trainset14_032015.pds.tax, cutoff=80)
```

```
#!/bin/bash -l
#SBATCH -J mothur
#SBATCH -o mothur.stdout
#SBATCH -e mothur.stderr
#SBATCH -n 5
#SBATCH -t 12:00:00
#SBATCH --nodes=1
#SBATCH --mem-per-cpu=23000
module load biokit
mothur mothur_task.txt
```

Output File Names:
rm -rf seqs_chimeras_filtered.pds.wang.taxonomy
rm -rf seqs_chimeras_filtered.pds.wang.tax.summary

rm -rf seqs_chimeras_filtered.pds.wang.flip.accnos

```
mothur > classify.seqs(fasta=seqs_chimeras_filtered1.fna,
reference=trainset14_032015.pds.fasta,
taxonomy=trainset14_032015.pds.tax, cutoff=80)
```

b. Remove mitochondrial, chroroplast, archea, eukaryotes and unknowns

```
mothur > remove.lineage(fasta=seqs_chimeras_filtered.fna,
taxonomy=seqs_chimeras_filtered.pds.wang.taxonomy,taxon=Chloroplast-
Mitochondria-unknown-Archaea-Eukarya)

mothur > remove.lineage(fasta=seqs_chimeras_filtered1.fna,
taxonomy=seqs_chimeras_filtered1.pds.wang.taxonomy,taxon=Chloroplast-
Mitochondria-unknown-Archaea-Eukarya)
```

assign taxonomy using HOMD database. Latest (2017-01-03) HOMD downloaded from ftp://www.homd.org/16S_rRNA_refseq/HOMD_16S_rRNA_RefSeq/

```
wget ftp://www.homd.org/16S_rRNA_refseq/HOMD_16S_rRNA_RefSeq/
HOMD_16S_rRNA_RefSeq_V14.5.qiime.taxonomy
wget ftp://www.homd.org/16S_rRNA_refseq/HOMD_16S_rRNA_RefSeq/
HOMD_16S_rRNA_RefSeq_V14.51.aligned.fasta
```

```
assign_taxonomy.py -i seqs_chimeras_filtered.pick.fna -r
HOMD_16S_rRNA_RefSeq_V14.51.aligned.fasta -t
HOMD_16S_rRNA_RefSeq_V14.5.qiime.taxonomy   -o assigned_taxonomy
```

```
#!/bin/bash -l
#SBATCH -J qiime_assign
#SBATCH -o qiime_assign.stdout
#SBATCH -e qiime_assign.stderr
#SBATCH -n 5
```

#SBATCH -t 20:00:00
#SBATCH --nodes=1
#SBATCH --mem-per-cpu=23000
module load qiime
assign_taxonomy.py -i seqs_chimeras_filtered.pick.fna -r HOMD_16S_rRNA_RefSeq_V14.51.aligned.fasta -t HOMD_16S_rRNA_RefSeq_V14.5.qiime.taxonomy -o assigned_taxonomy

assign_taxonomy.py -i seqs_chimeras_filtered1.pick.fna -r HOMD_16S_rRNA_RefSeq_V14.51.aligned.fasta -t HOMD_16S_rRNA_RefSeq_V14.5.qiime.taxonomy -o assigned_taxonomy1

Remove bacterial contaminants using blank and mock samples

usearch -usearch_global seqs_chimeras_filtered.pick.fna -db contaminant1.fasta -id 0.99 -strand plus --notmatched cleaned_seqs.fasta -threads 2

usearch -usearch_global seqs_chimeras_filtered1.pick.fna -db contaminant1.fasta -id 0.99 -strand plus --notmatched cleaned_seqs1.fasta -threads 2

usearch.sh

#!/bin/bash -l
#SBATCH -J usrch
#SBATCH -o urch.stdout
#SBATCH -e urch.stderr
#SBATCH -n 1
#SBATCH -t 2:00:00
#SBATCH --nodes=1
#SBATCH --mem-per-cpu=23000
usearch -usearch_global seqs_chimeras_filtered.pick.fna -db contaminant1.fasta -id 0.99 -strand plus --notmatched cleaned_seqs.fasta -threads 2

05:14 1.1Gb 100.0% Searching, 16.1% matched

Here on continue with cleaned_seqs.fasta

OTU picking

#!/bin/bash -l

#SBATCH -J otu
#SBATCH -o otu.stdout
#SBATCH -e otu.stderr
#SBATCH -n 1
#SBATCH -t 6:00:00
#SBATCH --nodes=1
#SBATCH --mem-per-cpu=23000

module load qiime

pick_otus.py -i cleaned_seqs.fasta -o otu
pick_otus.py -i cleaned_seqs1.fasta -o otu1

pick representative seq

pick_rep_set.py -i otu/cleaned_seqs_otus.txt -f cleaned_seqs.fasta -o representative_seqs.fna
pick_rep_set.py -i otu1/cleaned_seqs1_otus.txt -f cleaned_seqs1.fasta -o representative_seqs1.fna

align the representative sequences

align_seqs.py -i representative_seqs.fna -t core_alignment_SILVA123.fasta -o aligned/
align_seqs.py -i representative_seqs1.fna -t core_alignment_SILVA123.fasta -o aligned1/

## taxonomy assignment

assign_taxonomy.py -i representative_seqs.fna -r HOMD_16S_rRNA_RefSeq_V14.51.aligned.fasta -t HOMD_16S_rRNA_RefSeq_V14.5.qiime.taxonomy -o assigned_taxonomy_repre
assign_taxonomy.py -i representative_seqs1.fna -r HOMD_16S_rRNA_RefSeq_V14.51.aligned.fasta -t HOMD_16S_rRNA_RefSeq_V14.5.qiime.taxonomy -o assigned_taxonomy_repre1

## filter alignment
filter_alignment.py -i aligned/representative_seqs_aligned.fasta -o filtered_alignment/
filter_alignment.py -i aligned1/representative_seqs1_aligned.fasta -o

filtered_alignment1/

## building tree
make_phylogeny.py -i filtered_alignment/representative_seqs_aligned_pfiltered.fasta -o rep_phylo.tre
make_phylogeny.py -i filtered_alignment1/representative_seqs1_aligned_pfiltered.fasta -o rep_phylo1.tre

## make OTU table
make_otu_table.py -i otu/cleaned_seqs_otus.txt -t assigned_taxonomy_repre/representative_seqs_tax_assignments.txt -o otu_table.biom
make_otu_table.py -i otu1/cleaned_seqs1_otus.txt -t assigned_taxonomy_repre1/representative_seqs1_tax_assignments.txt -o otu_table1.biom

Filter out Mock and blank samples
filter_samples_from_otu_table.py -i otu_table.biom -o filtered_otu_table.biom --sample_id_fp ids.txt --negate_sample_id_fp
filter_samples_from_otu_table.py -i otu_table1.biom -o filtered_otu_table1.biom --sample_id_fp ids.txt --negate_sample_id_fp

Remove singletons
filter_otus_from_otu_table.py -i filtered_otu_table.biom -o otu_table_no_singletons.biom -n 2
filter_otus_from_otu_table.py -i filtered_otu_table1.biom -o otu_table_no_singletons1.biom -n 2

check summary (47 samples remaining)
biom summarize-table -i filtered_otu_table.biom
biom summarize-table -i filtered_otu_table1.biom

Normalize OTU table
module load qiime/1.9.1
normalize_table.py -i otu_table_no_singletons.biom -a CSS -o CSS_normalized_otu_table.biom
normalize_table.py -i otu_table_no_singletons1.biom -a CSS -o CSS_normalized_otu_table1.biom

Remove Blank and Mock samples from map

Here mock and blank samples are removed from updated_map.txt.

map_updated1_noMock.txt
map_updated_noMock.txt

There are many metrics but we use unweigted unifrac (most popular)

```
beta_diversity.py -i CSS_normalized_otu_table.biom -m
unweighted_unifrac -t rep_phylo.tre -o beta_div
beta_diversity.py -i CSS_normalized_otu_table1.biom -m
unweighted_unifrac -t rep_phylo1.tre -o beta_div1
```

Plot beta diversity

```
beta_diversity_through_plots.py -i otu_table_no_singletons.biom -o
bdiv_even8000/ -t rep_phylo.tre -m map.txt -e 8000
```

Adonis

```
compare_categories.py --method adonis -i ./beta_div/
unweighted_unifrac_CSS_normalized_otu_table.txt -m
map_updated_noMock.txt -c Treatment -o adonis_out

# r2 = 0.10 (0.001)

compare_categories.py --method adonis -i ./beta_div1/
unweighted_unifrac_CSS_normalized_otu_table1.txt -m
map_updated1_noMock.txt -c Treatment -o adonis_out1
# r2 = 0.14 (0.001)
```

PERMDISP

```
compare_categories.py --method permdisp -i ./beta_div/
unweighted_unifrac_CSS_normalized_otu_table.txt -m
map_updated_noMock.txt -c Treatment -o permdisp_out

#F Value = 0.78 (0.38)
```

```
compare_categories.py --method permdisp -i ./beta_div1/
unweighted_unifrac_CSS_normalized_otu_table1.txt -m
map_updated1_noMock.txt -c Treatment -o permdisp_out1

#F Value = 4.65 (0.04)
```

Alpha diversity using phyloseq

See phyloseq_plots.R for plots and also for following

```
#dicotomize smoking variable and then do following
ind_smk <- which(map2_mat$Smoking=="YES")
ind_na <- which(is.na(map2_mat$Smoking))
erich1 <- erich[-ind_na,]
map_sub <- map2_mat[-ind_na,]
t.test(erich1$Simpson[-ind_smk],erich1$Simpson[ind_smk])
t.test(erich1$Shannon[-ind_smk],erich1$Shannon[ind_smk])
t.test(erich1$Chao1[-ind_smk],erich1$Chao1[ind_smk])
t.test(erich1$Observed[-ind_smk],erich1$Observed[ind_smk])

# plot
library(ggplot2)
p <-
qplot(factor(map_sub$Smoking),erich1$Shannon,geom="boxplot",xlab
="Smoking",ylab="Alpha diversity (Shannon)")
Smoking = factor(map_sub$Smoking)
p + geom_boxplot(aes(fill = Smoking)) + geom_jitter()
```

```
erich <- estimate_richness(dat1, measures = c("Observed",
"Shannon", "Simpson", "Chao1"))

# perform t-test for alpha diversity between case and control
ind_k <- grep("K",rownames(erich))
t.test(erich$Simpson[-ind_k],erich$Simpson[ind_k]) # Simpson. No
significance.
t.test(erich$Shannon[-ind_k],erich$Shannon[ind_k]) # Shannon. No
```

```
significance
t.test(erich$Chao1[-ind_k],erich$Chao1[ind_k]) # Chao1. No
Significant!
t.test(erich$Observed[-ind_k],erich$Observed[ind_k]) # Observed.
No significant.


# t test of alpha between male and female
ind_sex <- which(map2_mat$Sex==“Male”)
t.test(erich$Observed[ind_sex],erich$Observed[-ind_sex]) # 0.07
t.test(log2(erich$Shannon[ind_sex]),log2(erich$Shannon[-
ind_sex])) # 0.02
t.test(log2(erich$Simpson[ind_sex]),log2(erich$Simpson[-
ind_sex])) # 0.03
t.test(erich$Chao1[ind_sex],erich$Chao1[-ind_sex]) # 0.10




# associate alpha diversity with clinical variables (only for
cases)
samples <- rownames(erich)
samples <- gsub("^.*?X","",samples) # remove X from beginning of
sample ids
rownames(erich) <- samples
erich <- erich[match(map2_mat[,1],rownames(erich)),] # order it
same as map2_mat

cor(erich$Observed[1:34],map2_mat$periapiocal_lesion_yes_no[1:34
]) # 0.32
cor(erich$Observed[1:34],map2_mat$X6_and_over_yes_no[1:34]) #[1]
0.1526286
cor(erich$Observed[1:34],map2_mat$X6.over_6_pocket[1:34]) #[1]
0.2042794
cor(erich$Observed[1:34],map2_mat$X4.5_mm_pocket[1:34]) #[1]
-0.1927096
cor(erich$Observed[1:34],map2_mat$X4.5mm_yes_no[1:34]) #[1]
-0.07994545
cor(erich$Observed[1:34],map2_mat$Infection_score[1:34]) #[1]
0.5503354
cor(erich$Observed[1:34],map2_mat$Deep_caries[1:34]) #[1]
0.4596935
cor(erich$Observed[1:34],map2_mat$Vertical_pockets[1:34]) #[1]
-0.2235755
cor(erich$Observed[1:34],map2_mat$Furkation_lesions[1:34]) #[1]
-0.245748
```

```
cor(erich$Observed[1:34],map2_mat$Periapical_lesions[1:34]) #[1]
0.5926242
cor(erich$Observed[1:34],map2_mat$periapiocal_lesion_yes_no[1:34
]) #[1] 0.3158256
cor(erich$Observed[1:34],map2_mat$Tooth_brushing[1:34]) #[1]
-0.512175
cor(erich$Observed,map2_mat$Age) #[1] 0.1673368

cor(erich$Chao1[1:34],map2_mat$periapiocal_lesion_yes_no[1:34])
# 0.32
cor(erich$Chao1[1:34],map2_mat$X6_and_over_yes_no[1:34]) #[1]
0.10
cor(erich$Chao1[1:34],map2_mat$X6.over_6_pocket[1:34]) #[1] 0.22
cor(erich$Chao1[1:34],map2_mat$X4.5_mm_pocket[1:34]) #[1] -0.17
cor(erich$Chao1[1:34],map2_mat$X4.5mm_yes_no[1:34]) #[1] 0.007
cor(erich$Chao1[1:34],map2_mat$Infection_score[1:34]) #[1] 0.57
cor(erich$Chao1[1:34],map2_mat$Deep_caries[1:34]) #[1] 0.48
cor(erich$Chao1[1:34],map2_mat$Vertical_pockets[1:34]) #[1]
-0.22
cor(erich$Chao1[1:34],map2_mat$Furkation_lesions[1:34]) #[1]
-0.24
cor(erich$Chao1[1:34],map2_mat$Periapical_lesions[1:34]) #[1]
0.59
cor(erich$Chao1[1:34],map2_mat$periapiocal_lesion_yes_no[1:34])
#[1] 0.32
cor(erich$Chao1[1:34],map2_mat$Tooth_brushing[1:34]) #[1] -0.57
cor(erich$Chao1,map2_mat$Age) # 0.11


cor(erich$Simpson[1:34],map2_mat$periapiocal_lesion_yes_no[1:34]
) # 0.24
cor(erich$Simpson[1:34],map2_mat$X6_and_over_yes_no[1:34]) #[1]
0.09
cor(erich$Simpson[1:34],map2_mat$X6.over_6_pocket[1:34]) #[1]
0.12
cor(erich$Simpson[1:34],map2_mat$X4.5_mm_pocket[1:34]) #[1]
-0.22
cor(erich$Simpson[1:34],map2_mat$X4.5mm_yes_no[1:34]) #[1] -0.18
cor(erich$Simpson[1:34],map2_mat$Infection_score[1:34]) #[1]
0.18
cor(erich$Simpson[1:34],map2_mat$Deep_caries[1:34]) #[1] 0.24
cor(erich$Simpson[1:34],map2_mat$Vertical_pockets[1:34]) #[1]
-0.02
cor(erich$Simpson[1:34],map2_mat$Furkation_lesions[1:34]) #[1]
0.26
cor(erich$Simpson[1:34],map2_mat$Periapical_lesions[1:34]) #[1]
0.24
```

```
cor(erich$Simpson[1:34],map2_mat$periapiocal_lesion_yes_no[1:34]
) #[1] 0.24
cor(erich$Simpson[1:34],map2_mat$Tooth_brushing[1:34]) #[1]
-0.34
cor(erich$Simpson,map2_mat$Age) #[1] -0.02


# others are similar here


# tooth-brushing analysis
library(ggplot2)
p <-
qplot(factor(map2_mat$Tooth_brushing[1:34]),erich$Chao1[1:34],ge
om="boxplot",xlab="Tooth brushing per day",ylab="Alpha diversity
(Chao1)")
Tooth_brushing = factor(map2_mat$Tooth_brushing[1:34])
p + geom_boxplot(aes(fill = Tooth_brushing)) + geom_jitter()


# shannon
library(ggplot2)
p1 <-
qplot(factor(map2_mat$Tooth_brushing[1:34]),erich$Shannon[1:34],
geom="boxplot",xlab="Tooth brushing per day",ylab="Alpha
diversity (Shannon)")
p1 + geom_boxplot(aes(fill = Tooth_brushing)) + geom_jitter()


# Simpson
library(ggplot2)
p2 <-
qplot(factor(map2_mat$Tooth_brushing[1:34]),erich$Simpson[1:34],
geom="boxplot",xlab="Tooth brushing per day",ylab="Alpha
diversity (Simpson)")
p2 + geom_boxplot(aes(fill = Tooth_brushing)) + geom_jitter()




tiff(file = “tooth_box.tiff", width = 3200, height = 3200, units =
"px", res = 300) # this is for 4 by 4 inch and 300 dbi
p <-
qplot(factor(map2_mat$Tooth_brushing[1:34]),erich$Chao1[1:34],ge
om="boxplot",xlab="Tooth brushing per day",ylab="Alpha diversity
(Chao1)")
```

```
Tooth_brushing = factor(map2_mat$Tooth_brushing[1:34])
p + geom_boxplot(aes(fill = Tooth_brushing)) + geom_jitter()
#### "tooth_box.tiff"
dev.off()


postscript("tooth_box.eps", width = 480, height = 480)
p <-
qplot(factor(map2_mat$Tooth_brushing[1:34]),erich$Chao1[1:34],ge
om="boxplot",xlab="Tooth brushing per day",ylab="Alpha diversity
(Chao1)")
Tooth_brushing = factor(map2_mat$Tooth_brushing[1:34])
p + geom_boxplot(aes(fill = Tooth_brushing)) + geom_jitter()
#### "tooth_box.tiff"
dev.off()


# also fit linear model to get statistics score and value
summary(lm(log2(erich$Chao1[1:34])~map2_mat$Tooth_brushing[1:34]))
# Adjusted R2 = 34% of the variation in Chao1 is explained tooth
brushing frequency with P-value of 0.0002


# check if lm was valid by plotting residuals
fit <- lm(log2(erich$Chao1[1:34])~map2_mat$Tooth_brushing[1:34])
qqnorm(resid(fit)) ## fair enough
qqline(resid(fit))
```

# periapical lesions

```
Periapical_lesions = factor(map2_mat$Periapical_lesions[1:34])
qplot(factor(map2_mat$Periapical_lesions[1:34]),erich$Chao1[1:34
],colour=Periapical_lesions,xlab="Periapical
lesions",ylab="Alpha diversity (Chao1)")
```

# periapical lesions yes or no

```
Periapiocal_lesion_yes_no =
factor(map2_mat$periapiocal_lesion_yes_no[1:34])
x <-
qplot(Periapiocal_lesion_yes_no,erich$Chao1[1:34],geom="boxplot"
```

```
,xlab="Periapiocal lesion yes no",ylab="Alpha diversity
(Chao1)")
x + geom_boxplot(aes(fill = Periapiocal_lesion_yes_no)) +
geom_jitter()


# also fit linear model to get statistics score and value
summary(lm(log2(erich$Chao1[1:34])~map2_mat$periapiocal_lesion_yes_n
o[1:34]))
# 7% of the variation in Chao1 is explained by periapoical
lesions with P-value of 0.07


# check if lm was valid by plotting residuals
fit1 <-
lm(log2(erich$Chao1[1:34])~map2_mat$periapiocal_lesion_yes_no[1:34])
qqnorm(resid(fit1)) ## fair enough
qqline(resid(fit1))



# Deep caries
deep_caries = factor(map2_mat$Deep_caries[1:34])
qplot(deep_caries,erich$Chao1[1:34],colour=deep_caries,xlab="Dee
p caries",ylab="Alpha diversity (Chao1)")



# X6 pocket
x6_pocket = factor(map2_mat$X6.over_6_pocket[1:34])
qplot(x6_pocket,erich$Chao1[1:34],colour=x6_pocket,xlab="X6
pockets",ylab="Alpha diversity (Chao1)")


# X6 pocket yes no
x6_pocket_yes_no = factor(map2_mat$X6_and_over_yes_no[1:34])
Y <-
qplot(x6_pocket_yes_no,erich$Chao1[1:34],geom="boxplot",xlab="X6
pockets yes no",ylab="Alpha diversity (Chao1)")
Y + geom_boxplot(aes(fill = x6_pocket_yes_no)) + geom_jitter()

summary(lm(log2(erich$Chao1[1:34])~map2_mat$X6_and_over_yes_no[1:34])
) # not significant
```

differential abundance analysis
for case-control

```
library(DESeq2)
diagdds_case_control = phyloseq_to_deseq2(dat2, ~ Treatment) ##
see phyloseq_plots.R for details

# calculate geometric means prior to estimate size factors
gm_mean = function(x, na.rm=TRUE){
  exp(sum(log(x[x > 0]), na.rm=na.rm) / length(x))
}
geoMeans = apply(counts(diagdds_case_control), 1, gm_mean)
diagdds_case_control = estimateSizeFactors(diagdds_case_control,
geoMeans = geoMeans)
diagdds_case_control = DESeq(diagdds_case_control,
fitType="local")


# test result table
res_case_control = results(diagdds_case_control)
res_case_control = res_case_control[order(res_case_control$padj,
na.last=NA), ]
alpha = 0.01
sigtab_case_control = res_case_control[(res_case_control$padj <
alpha), ]
sigtab_case_control = cbind(as(sigtab_case_control,
"data.frame"), as(tax_table(dat2)
[rownames(sigtab_case_control), ], "matrix"))
head(sigtab_case_control)

colnames(sigtab_case_control)[7:13] <-
c("Kingdom","Phylum","Class","Order","Family","Genus","Species")
library(xlsx)
write.xlsx(sigtab_case_control, file="sigtab_case_control.xlsx",
sheetName="Sheet1",row.names=FALSE)


ps.
> res_case_control
log2 fold change (MAP): Treatment Control vs Case
Wald test p-value: Treatment Control vs Case
DataFrame with 261 rows and 6 columns

# this means that negative fold change means the abundance is low in
Control or high in case
```

for tooth-brush groups

```
library(DESeq2)
colnames(dat2@otu_table)==row.names(dat2@sam_data)
dat2_brush <- dat2
dat2_brush@otu_table <- dat2_brush@otu_table[,-
which(is.na(dat2_brush@sam_data$Tooth_brushing))]
dat2_brush@sam_data <- dat2_brush@sam_data[-
which(is.na(dat2_brush@sam_data$Tooth_brushing)),]
dat2_brush@sam_data$Tooth_brushing  <-
factor(dat2_brush@sam_data$Tooth_brushing)
diagdds_brush = phyloseq_to_deseq2(dat2_brush, ~ Tooth_brushing)


# calculate geometric means prior to estimate size factors
gm_mean = function(x, na.rm=TRUE){
  exp(sum(log(x[x > 0]), na.rm=na.rm) / length(x))
}
geoMeans = apply(counts(diagdds_brush), 1, gm_mean)
diagdds_brush = estimateSizeFactors(diagdds_brush, geoMeans =
geoMeans)
diagdds_brush = DESeq(diagdds_brush, test="LRT",reduced=~ 1)


# test result table
res_brush = results(diagdds_brush)
res_brush = res_brush[order(res_brush$padj, na.last=NA), ]
alpha = 1
sigtab_brush = res_brush[(res_brush$padj < alpha), ]
sigtab_brush = cbind(as(sigtab_brush, "data.frame"),
as(tax_table(dat2)[rownames(sigtab_brush), ], "matrix"))
colnames(sigtab_brush)[7:13] <-
c("Kingdom","Phylum","Class","Order","Family","Genus","Species")
library(xlsx)
write.xlsx(sigtab_brush, file="sigtab_brush.xlsx",
sheetName="Sheet1",row.names=TRUE)


### Daa plot tooth brushing

plot.daa.brush <- dat2

imp_otu <- c("denovo469","denovo1174","denovo703")

imp.ind <- match(imp_otu,rownames(plot.daa.brush@otu_table))

plot.daa.brush@otu_table <- plot.daa.brush@otu_table[imp.ind,]
```

```
plot.daa.brush@tax_table <- plot.daa.brush@tax_table[imp.ind,]
rownames(plot.daa.brush@otu_table) <-
c("Fusobacteria","Fusobacteria","Bacteroidetes")
rownames(plot.daa.brush@tax_table) <-
c("Fusobacteria","Fusobacteria","Bacteroidetes")

df.brush.daa.imp <- as.data.frame(t(plot.daa.brush@otu_table))

sample.dat <- plot.daa.brush@sam_data
sample.dat <-
sample.dat[match(rownames(df.brush.daa.imp),sample.dat$X.SampleID),] #
same order
rm.na <- which(is.na(sample.dat$Tooth_brushing))

df.brush.daa.imp <- df.brush.daa.imp[-rm.na,]
sample.dat <- sample.dat[-rm.na,]
df.brush.daa.imp$Tooth_brushing <- sample.dat$Tooth_brushing
df.brush.daa.imp$Tooth_brushing <-
as.character(df.brush.daa.imp$Tooth_brushing)
colnames(df.brush.daa.imp) <-
c("Fusobacteria1","Fusobacteria2","Bacteroidetes","Tooth_brushin
g")

library(reshape2)
df.long <- melt(df.brush.daa.imp, id.vars="Tooth_brushing")
colnames(df.long)[3] <- "Abundance"


p2 <- ggplot(df.long,
aes(x=factor(Tooth_brushing),y=Abundance,fill=factor(Tooth_brush
ing)))+
  geom_boxplot() + labs(title=" ") +facet_wrap(~variable)
p2 + xlab("Tooth brushing habit"). ###
"Tooth_brush_daa_plot.png"




tiff(file = "Tooth_brush_daa_plot.tiff", width = 3200, height =
3200, units = "px", res = 300) # this is for 4 by 4 inch and 300 dbi
p2 <- ggplot(df.long,
aes(x=factor(Tooth_brushing),y=Abundance,fill=factor(Tooth_brush
ing)))+
  geom_boxplot() + labs(title=" ") +facet_wrap(~variable)
p2 + xlab("Tooth brushing habit")   # ###
"Tooth_brush_daa_plot.tiff"
dev.off()
```

```
postscript("Tooth_brush_daa_plot.eps", width = 480, height =
480)
p2 <- ggplot(df.long,
aes(x=factor(Tooth_brushing),y=Abundance,fill=factor(Tooth_brush
ing)))+
  geom_boxplot() + labs(title=" ") +facet_wrap(~variable)
p2 + xlab("Tooth brushing habit”)   # ###
“Tooth_brush_daa_plot.tiff”
dev.off()
```

```
## DAA results at phylum label

dat2_brush

a <- as.data.frame(dat2_brush@otu_table)
b <- as.data.frame(dat2_brush@tax_table)

# rm NA Rank2 (missing phylas)
ind <- which(is.na(b$Rank2))

a <- a[-ind,]
b <- b[-ind,]


# number of unique phylas
length(unique(b$Rank2)) # 11


# name the row names as the phylums
a <- as.matrix(a)
rownames(a) <- b$Rank2



# summarise by phyla
summarized_a <- t(sapply(by(a,rownames(a),colSums),identity))

# sample.labels
c <- as.data.frame(dat2_brush@sam_data)
```

```
# DESeq2
library(DESeq2)
samples <- as.data.frame(c$Tooth_brushing)
colnames(samples) <- “Tooth_brushing”
dds <- DESeqDataSetFromMatrix(countData=summarized_a, colData=samples,
design=~Tooth_brushing)
ds <- DESeq(dds)

res <- as.data.frame(results(ds))
res <- res[order(res$padj,decreasing=FALSE),]. # the Fuso is now not
in the top. Summarizing OTUs to phyla level does not seem a good idea.
write.xlsx(res, file=“sigtab_brush_phyla.xlsx",
sheetName="Sheet1",row.names=TRUE)
```

for x4.5mm_yes_no.

```
library(DESeq2)

# subset phyloseq object to contain only cases
map_phylo_obj <- dat2@sam_data
logi_group <- rep(TRUE,nrow(map_phylo_obj))
logi_group[grep("K",map_phylo_obj$X.SampleID)] = FALSE
dat2_cases <- prune_samples(logi_group,dat2)

diagdds_x4.5 = phyloseq_to_deseq2(dat2_cases, ~ X4.5mm_yes_no)

# calculate geometric means prior to estimate size factors
gm_mean = function(x, na.rm=TRUE){
  exp(sum(log(x[x > 0]), na.rm=na.rm) / length(x))
}
geoMeans = apply(counts(diagdds_x4.5), 1, gm_mean)
diagdds_x4.5 = estimateSizeFactors(diagdds_x4.5, geoMeans =
geoMeans)
diagdds_x4.5 = DESeq(diagdds_x4.5, fitType="local")


# test result table
res_x4.5 = results(diagdds_x4.5)
res_x4.5 = res_x4.5[order(res_x4.5$padj, na.last=NA), ]
alpha = 1
sigtab_x4.5 = res_x4.5[(res_x4.5$padj < alpha), ]
sigtab_x4.5 = cbind(as(sigtab_x4.5, "data.frame"),
as(tax_table(dat2_cases)[rownames(sigtab_x4.5), ], "matrix"))
head(sigtab_x4.5)
```

for x6_yes_no.

```
diagdds_x6 = phyloseq_to_deseq2(dat2_cases, ~
X6_and_over_yes_no)
geoMeans = apply(counts(diagdds_x6), 1, gm_mean)
diagdds_x6 = estimateSizeFactors(diagdds_x6, geoMeans =
geoMeans)
diagdds_x6 = DESeq(diagdds_x6, fitType="local")
# test result table
res_x6 = results(diagdds_x6)
res_x6 = res_x6[order(res_x6$padj, na.last=NA), ]
alpha = 1
sigtab_x6 = res_x6[(res_x6$padj < alpha), ]
sigtab_x6 = cbind(as(sigtab_x6, "data.frame"),
as(tax_table(dat2_cases)[rownames(sigtab_x6), ], "matrix"))
```

for periapiocal

```
diagdds_peri = phyloseq_to_deseq2(dat2_cases, ~
periapiocal_lesion_yes_no)
geoMeans = apply(counts(diagdds_peri), 1, gm_mean)
diagdds_peri = estimateSizeFactors(diagdds_peri, geoMeans =
geoMeans)
diagdds_peri = DESeq(diagdds_peri, fitType="local")
# test result table
res_peri = results(diagdds_peri)
res_peri = res_peri[order(res_peri$padj, na.last=NA), ]
alpha = 1
sigtab_peri = res_peri[(res_peri$padj < alpha), ]
sigtab_peri = cbind(as(sigtab_peri, "data.frame"),
as(tax_table(dat2_cases)[rownames(sigtab_peri), ], "matrix"))
```

plot DAA otus

```
daa_res_all <- res_case_control[(res_case_control$padj < 1), ]
daa_res_all <- cbind(as(daa_res_all, "data.frame"), as(tax_table(dat2)[rownames(daa_res_all), ],
"matrix"))
imp_otu_ind <- c(200,70,83,37,54,66)
daa_res_imp <- daa_res_all[imp_otu_ind,] # this is the top table for imp bacteria
```

```
library(xlsx)
```

```
write.xlsx(daa_res_imp, file="daa_res_imp.xlsx",
sheetName="Sheet1",row.names=FALSE)



plot.dat3 <- dat2
ind_imp <- match(rownames(daa_res_imp),rownames(plot.dat3@otu_table))



plot.dat3@otu_table <- plot.dat3@otu_table[ind_imp,]
plot.dat3@tax_table <- plot.dat3@tax_table[ind_imp,]
rownames(plot.dat3@otu_table) <- c("Prevotella
intermedia","Fusobacterium nucleatum_subsp._vincentii","Porphyromonas
gingivalis","Streptocccaceae NA" ,"Streptocccaceae
NA","Streptocccaceae NA")
rownames(plot.dat3@tax_table) <- c("Prevotella
intermedia","Fusobacterium nucleatum_subsp._vincentii","Porphyromonas
gingivalis","Streptocccaceae NA" ,"Streptocccaceae
NA","Streptocccaceae NA")


df_imp <- as.data.frame(t(plot.dat3@otu_table))

trtmt <- rownames(df_imp)
ind_k <- grep("K",trtmt)
trtmt[ind_k] <- "Control"
trtmt[-ind_k] <- "Case"
df_imp <- cbind(Treatment=trtmt,df_imp)


library(reshape2)
df_imp_long <- melt(df_imp, id.vars="Treatment")


p2 <- ggplot(df_imp_long,
aes(x=factor(Treatment),y=value,fill=factor(Treatment)))+
  geom_boxplot() + labs(title=" ") +facet_wrap(~variable)
p2 + xlab(“Treatment”)
```
